# Editorial: Biomass innovations for a renewable and sustainable chemicals industry

**DOI:** 10.3389/fchem.2026.1939549

**Published:** 2026-07-27

**Authors:** Fabrizio Olivito, Goldie Oza, Pravin Jagdale

**Affiliations:** 1 Department of Environmental Engineering, University of Calabria, Arcavacata, Italy; 2 Centro de Investigation y Desarollo Tecnologico en Electroquimica (CIDETEQ), Pedro Escobedo, Mexico; 3 BHUMI Pvt Ltd., Mumbai, India

**Keywords:** biomass valorization, circular bioeconomy, green chemistry, renewable chemicals, sustainable materials

The transition towards a low-carbon and circular economy is driving a profound transformation of the chemicals industry, where sustainability is no longer limited to replacing fossil resources with renewable alternatives, but increasingly relies on redesigning processes, materials, and value chains according to the principles of green chemistry. In this context, renewable biological resources, and biomass in particular, offer unique opportunities for producing chemicals, fuels, advanced materials, and bio-based products while promoting resource efficiency and circularity. However, unlocking their full potential requires integrated approaches that combine innovative conversion technologies, efficient resource utilization, waste minimization, and environmentally responsible process design.

To address these challenges, the Research Topic *Biomass Innovations for a Renewable and Sustainable Chemicals Industry* was conceived to highlight recent advances demonstrating how renewable biological resources can contribute to the development of sustainable technologies across different areas of chemistry and engineering. Although the contributions collected in this Research Topic address diverse applications, they converge towards a common objective: maximizing the value of renewable biological resources through innovative approaches that improve process efficiency, reduce environmental impacts, and promote the transition towards a circular bioeconomy.

A common theme emerging from this Research Topic is the progressive diversification of renewable-resource valorization strategies. Traditionally regarded as an alternative feedstock for producing fuels and commodity chemicals, biomass is increasingly being explored as a versatile source of advanced functional materials, renewable energy carriers, and biologically derived products. This evolution reflects a broader transformation in sustainable chemistry, where renewable resources are no longer viewed simply as substitutes for fossil carbon but as enabling platforms for innovative technologies capable of addressing environmental and societal challenges.

Catalysis remains a cornerstone of this transition. The development of highly efficient heterogeneous catalytic systems capable of selectively converting biomass-derived platform molecules while facilitating catalyst recovery and reuse represents a key enabling strategy for greener chemical manufacturing. In this context, Alorfi and Abu-Hamdeh (2026) developed a sulfonated graphene oxide/MnFe_2_O_4_ magnetic nanocomposite for the efficient etherification of 5-hydroxymethylfurfural into five-ethoxymethylfurfural, combining high catalytic activity, excellent selectivity, and straightforward magnetic recovery. Their work exemplifies how rational catalyst design can simultaneously improve reaction efficiency, catalyst recyclability, and process sustainability, addressing key requirements for future biomass conversion technologies.

Beyond catalytic biomass conversion, renewable feedstocks are increasingly recognized as valuable precursors for advanced functional materials. Agricultural residues, traditionally considered low-value waste streams, are increasingly being transformed into high-performance carbon materials for energy storage applications. Navarro Di Mari et al. (2026) demonstrated that garlic-braid agricultural waste can be converted into activated biochar with a hierarchical porous structure suitable as a sulfur host for lithium–sulfur batteries. Beyond the excellent electrochemical performance achieved, this work highlights how biomass-derived materials can simultaneously promote waste valorization, resource efficiency, and the development of sustainable energy technologies.

The scope of biomass valorization is further broadened through the integration of biotechnology and circular economy principles. Tropea et al. (2025) explored the use of the naturally produced microbial pigment violacein as an environmentally friendly probe molecule for recycled paper-based surface-enhanced Raman scattering (SERS) substrates. By combining microbial biosynthesis with recycled paper platforms, the study demonstrates how bio-based products can successfully replace conventional synthetic materials in advanced analytical technologies, opening new opportunities for sustainable sensing applications in environmental monitoring, food safety, diagnostics, and cultural heritage preservation.

Complementing these approaches, the efficient recovery of energy from organic waste remains a fundamental component of sustainable resource management. Zeng et al. (2026) investigated methane production and microbial community dynamics in bioreactor landfills, providing valuable insights into the relationships between leachate composition, microbial ecology, and anaerobic digestion performance. Their findings improve our understanding of the biological mechanisms governing methane production and suggest strategies for enhancing process stability and renewable energy recovery from municipal solid waste.

Although these studies address different scientific questions and technological applications, they collectively illustrate the diversity and multidisciplinary nature of contemporary research on renewable biological resources. Taken together, these contributions illustrate that biomass is no longer viewed solely as an alternative feedstock for renewable fuels or commodity chemicals. Instead, it is increasingly recognized as a versatile platform for developing catalysts, functional materials, analytical technologies, and biological processes that support a more sustainable use of natural resources. Despite their different scopes, all contributions share the fundamental principles of modern green chemistry, including the use of renewable resources, the valorization of agricultural and municipal residues, the design of recyclable materials and processes, and the reduction of environmental impacts through innovation.

The multidisciplinary nature of the contributions further highlights that progress towards a renewable and sustainable chemicals industry depends on the integration of chemistry, materials science, biotechnology, environmental engineering, and process design. Such interdisciplinary approaches will be essential for overcoming the remaining challenges associated with process scale-up, lifecycle assessment, techno-economic feasibility, and industrial implementation.

Collectively, the articles published in this Research Topic demonstrate that the transition towards a sustainable chemicals industry is no longer driven simply by replacing fossil-derived feedstocks with biomass. Rather, it is being shaped by integrated approaches that combine catalysis, advanced materials, biotechnology, and environmental engineering to maximize resource efficiency and promote circularity. Looking ahead, further progress will depend on strengthening interdisciplinary collaborations and integrating sustainability assessment, life-cycle thinking, and techno-economic evaluation from the earliest stages of technology development. Such holistic approaches will be essential to translate laboratory-scale innovations into scalable, economically viable, and environmentally responsible solutions. Ultimately, continued innovation in renewable-resource utilization will play a pivotal role in accelerating the transition towards a circular, climate-neutral, and resilient chemicals industry.

